# Confidence and Care: Understanding Staff Needs in Supporting Families Through Pregnancy Loss in Gynaecological Settings

**DOI:** 10.1155/nrp/2981752

**Published:** 2026-07-19

**Authors:** Suzanne Heaney, Áine Aventin, Martina Galeotti

**Affiliations:** ^1^ School of Nursing and Midwifery, Queen’s University, Belfast, UK, qub.ac.uk

**Keywords:** education, gynaecology, health and wellbeing, healthcare professionals, mixed-methods, pregnancy loss, training

## Abstract

**Aim:**

To explore healthcare professionals’ confidence, skills and organisational support needs in providing pregnancy loss care within gynaecological settings in Northern Ireland.

**Design:**

This is a mixed‐methods, exploratory study.

**Methods:**

An anonymous online survey was completed by 18 healthcare professionals. Quantitative data were collected using the *Perinatal Bereavement Care Confidence Scale* and analysed with descriptive statistics. Open‐ended responses were analysed through qualitative content analysis.

**Results:**

The majority (83%) of participants acknowledged that pregnancy loss is a traumatic event for women. However, only 11% of participants felt well prepared to provide bereavement support. Organisational and environmental barriers were perceived to impede compassionate practice, and the majority of participants (94%) believed continuing bereavement education is needed. Grief counselling skills were highlighted as a particular training need. Analysis of the qualitative data generated four overarching categories reflecting healthcare professionals’ perspectives on pregnancy loss care within gynaecological settings: (1) creating safe and compassionate spaces, (2) systemic and structural pressures undermine care, (3) the need for skilled, confident and supported staff and (4) building organisational commitment to bereavement care.

**Conclusion:**

Healthcare professionals aspire to deliver dignified, person‐centred care following pregnancy loss but face systematic and educational barriers. Increasing organisational support as well as investment in staffing levels, bereavement training and access to emotional support are essential for the provision and sustainability of high‐quality bereavement care and staff wellbeing.

**Implications for the Profession:**

Embedding bereavement education, reflective practice and dedicated bereavement care environments within gynaecological services could foster confidence and consistency of bereavement care for pregnancy loss in this setting.

**Patient or Public Contribution:**

An advisory group comprising clinicians, bereavement support organisations and women with lived experience contributed to this study.

**Reporting Method:**

This study adheres to the GRAMMS checklist for mixed‐methods reporting.

## 1. Background

Pregnancy loss is a profound event that affects a significant proportion of individuals and families worldwide [1]. It encompasses different clinical outcomes including early biochemical pregnancy loss, miscarriage, molar pregnancy, ectopic pregnancy, termination of pregnancy for medical reasons (TFMRs) and stillbirth. Although frequently grouped together under the umbrella term ‘pregnancy loss’, they differ substantially in aetiology, gestational timing, diagnostic pathway and clinical management. Miscarriage is the most common pregnancy complication globally, with an estimated 23 million miscarriages annually [1]. In the United Kingdom (UK), 10%–20% of clinically recognised pregnancies end in miscarriage; approximately 1 in 90 pregnancies are ectopic each year; the stillbirth rate in England and Wales in 2024 was 3.9 per 1000 births [2], and in Scotland, it was 3.5 per 1000 births [3, 4]. In 2022, 3397 pregnancies in Great Britain resulted in TFMRs [3, 5]. In Northern Ireland (NI), the stillbirth rate in 2023 was 3.1 per 1000 total births (*n* = 60) [6], although routine surveillance data for other types of pregnancy loss are limited; there are no published TFMR data, and estimates suggest 3000–5000 miscarriages annually, of which 1000–3000 result in hospital admission [7].

The consequences of pregnancy loss extend beyond physical morbidity and can include psychological, emotional, relational and social impacts that endure long after the event [8–10]. Documented outcomes for women include post‐traumatic stress disorder, complicated grief, depression and relationship difficulties [11, 12]. A meta‐analysis reported clinically significant post‐traumatic stress symptoms in 29% of women at 1 month postloss and 18% at 9 months following miscarriage or ectopic pregnancy [13]. Partners can also be affected, often experiencing anxiety, suppressed grief and relationship strain [14].

Care pathways for pregnancy loss vary according to gestation, clinical presentation and local service configuration, and women may receive care across maternity units, early pregnancy assessment units (EPUs), emergency departments or gynaecology wards [15]. In NI and Ireland, recent research has highlighted that care experiences within gynaecology settings are often negative, characterised by the lack of privacy, poor communication and environments not conducive to bereavement care [7, 16].

Previous research in NI over two decades ago reported that gynaecology nurses felt inadequately prepared to support women experiencing pregnancy loss [17], with no updated evidence published since. In more recent work, many healthcare professionals (HCPs), regardless of setting, have continued to report feeling underprepared to provide bereavement care for pregnancy loss [18–20]. Reasons cited include insufficient training, limited clinical exposure and the emotional demands associated with these encounters. HCPs play a pivotal role in supporting women and families [21, 22]. Compassion, good communication, clear information and sensitive support are consistently identified as protective factors that help mitigate psychological distress and promote adjustment [9, 23], whereas poor communication, lack of privacy and insufficient acknowledgement of the loss can exacerbate trauma and contribute to disenfranchised grief [24]. Much of this evidence, however, is derived from studies of women’s experiences, and comparatively less is known about the needs and perspectives of HCPs providing this care. This study aimed to explore the needs of HCPs working in gynaecology settings in NI in providing bereavement care for pregnancy loss.

## 2. Methods

### 2.1. Study Design

This study employed a mixed‐methods, exploratory design. The study population comprised HCPs working in gynaecology settings in NI who cared for parents experiencing pregnancy loss as part of their role. Participants completed a short, anonymous survey.

### 2.2. Study Setting

This study focussed specifically on gynaecology settings to achieve a homogenous service context. In NI, gynaecology wards represent the most consistent and dominant site of pregnancy loss care, whereas EPUs are outpatient, early gestation diagnosis services with variable access, referral pathways and staff configurations across the region. EPUs were therefore excluded to avoid introducing service heterogeneity unlikely to be comparable within the scope of this study. Similarly, maternity units and emergency departments were excluded because they operate within distinct organisational, staffing and clinical contexts. Women experiencing pregnancy loss in these settings may present at different gestations, follow different care pathways and be supported by HCPs with different roles, training and resources. Restricting the study to gynaecology settings enabled exploration of a more homogenous service context and identification of setting‐specific educational and organisational support needs. NI is a jurisdiction with distinct service structures, commissioning and policy context comparable with the rest in the UK.

### 2.3. Participant Recruitment and Selection

#### 2.3.1. Personal and Public Involvement

An advisory group was established to guide and inform research design and decisions to ensure the voices of those most affected were represented. The group was comprised of clinicians (including a nurse, midwife and gynaecologist), representatives from bereavement support organisations and women with lived experience. Members contributed to the design and quality assurance of the study, including agreeing the study objectives, survey materials and dissemination methods. Participation was voluntary with members able to withdraw at any time. No data were collected from advisory group members; their role was to provide expertise based on professional and/or personal experience.

#### 2.3.2. Recruitment and Sampling

Eligible participants were HCPs aged over 18, currently practising in NI, who had provided care to women and families experiencing pregnancy loss within a gynaecology setting at some point during the previous 5 years. Retired staff, student HCPs and those working outside NI were excluded.

Pregnancy loss care within gynaecology services is provided across the five Health and Social Care Trusts in NI. Given the exploratory nature of the study and project timeline, recruitment was undertaken through professional and voluntary networks rather than through individual Health and Social Care Trust research governance processes. This approach enabled access to HCPs working across a range of clinical settings but resulted in a self‐selecting sample.

Recruitment used purposive and snowball sampling through professional and voluntary networks (e.g. Royal College of Nursing, Royal College of Midwives, the NI Pregnancy Loss Research and Policy Network, the NI Pregnancy Loss Charity Collective and the Miscarriage Association). An infographic poster with a QR code directed potential participants to the participant information sheet and survey. No incentives were offered.

Despite broad dissemination through professional and voluntary networks, recruitment proved challenging. We discussed this with our advisory group and workforce pressure was raised as the main issue why HCPs were not engaging in research activities. To try and address this, on the advice of the advisory group, we promoted the short survey length and the flexibility to not have to complete it in one go. Despite repeated reminders and the survey remaining open longer than planned (March–September 2025), participation remained lower than anticipated.

### 2.4. Data Collection

Data were collected via an anonymous online survey (Microsoft Forms). The survey comprised demographic items (*n* = 9) and the Perinatal Bereavement Care Confidence Scale (PBCCS) [25] (*n* = 43 statements). In addition to the PBCCS, a further *n* = 12 questions were asked, including three open‐ended questions to capture participants’ perspectives on barriers and facilitators to supporting women experiencing pregnancy loss in gynaecology settings (Table 1). These questions were developed in consultation with the study’s advisory group, ensuring their relevance to local practice contexts and alignment with the study aim.

**TABLE 1:** Qualitative questions.

1. In your opinion what does good pregnancy loss care look like in a gynaecological setting?
2. What do you think are the challenges in providing good pregnancy loss care in your setting?
3. What training/support do you need in order to be able to provide good pregnancy loss care? (Please be specific to topics, skills or elements of practice)

The PBCCS was selected as the primary instrument to assess HCPs’ confidence in providing bereavement care within gynaecology services. The PBCCS was developed in collaboration with midwives in an Irish maternity care service, making it contextually relevant to perinatal loss in similar healthcare systems [25]. The scale has demonstrated good inter‐rater reliability and face validity, supporting its suitability as a psychometrically sound tool. Importantly, the PBCCS captures multiple dimensions of bereavement care, including practical, emotional, cultural and legal aspects that align with the present study’s aim of exploring the current needs of HCPs working in gynaecology settings in NI.

The PBCCS includes four subscales: Bereavement Support Knowledge, Bereavement Support Skills, Self‐Awareness and Organisational Support. Responses were rated on a five‐point Likert scale (1 = Strongly Disagree to 5 = Strongly Agree). The PBCCS demonstrated good psychometric properties in the original validation (Cronbach’s *α* = 0.663–0.855) [25]. For inclusivity in this study, the word ‘midwives’ was amended to ‘HCPs’ to reflect the multidisciplinary sample.

### 2.5. Data Analysis

Survey data were downloaded into Microsoft Excel for descriptive analysis. Open‐ended responses were analysed using qualitative content analysis [26], supported by NVivo 12. We followed Bengtsson’s [26] approach of decontextualisation, recontextualisation, categorisation and compilation. Each response was read through and key meaning units highlighted (decontextualisation). The meaning units were checked against the full dataset to ensure nothing relevant was missed (recontextualisation). Research team discussions were held at this stage to review coding decisions, and themes were cross‐checked collaboratively to enhance consistency and ensure shared interpretation. Together, codes were grouped into subcategories and overarching categories (categorisation). Finally, findings were then compiled together for reporting (compilation). Throughout the analysis, the research team engaged in reflexive consideration of their professional backgrounds and experiences in pregnancy loss care, acknowledging how these perspectives might shape interpretation and decision‐making.

### 2.6. Ethical Considerations

Ethical approval was obtained from Queen’s University Belfast Faculty of Medicine, Health and Life Sciences Research Ethics Committee (Ref: MHLS 24_143). Surveys were anonymous with responses assigned unique identifiers (e.g. HCP01, HCP02) in chronological order of their submission. Data were stored securely in line with GDPR and will be retained for 5 years. Only the research team had access. Informed consent was obtained electronically prior to survey completion. Participants could withdraw at any stage, though submitted responses could not be removed due to anonymity.

## 3. Findings

### 3.1. Participants

Eighteen individuals completed the survey. Sociodemographic information of the participants is presented in Table 2. All 18 participants were female. Eleven out of the 18 had over 10 years clinical experience, with only one participant having less than 1 year of experience. The professions represented included healthcare assistant, nurse, midwife and doctor.

**TABLE 2:** Sociodemographic information of the participants (*n* = 18).

	*n* (%)
Gender	
Female	18 (100%)
Age (years)	
18–29	2 (11.1%)
30–39	4 (22.2%)
40–49	9 (50%)
50–59	3 (16.7%)
Education level	
Certificate	1 (5.6%)
Diploma	3 (16.7%)
Higher diploma	4 (22.2%)
Bachelors’ degree	8 (44.4%)
Master’s degree	1 (5.6%)
Doctorate	0 (0.0%)
Other	1 (5.6%)
Profession	
Healthcare assistant	3 (16.7%)
Nurse	6 (33.3%)
Midwife	4 (22.2%)
Doctor	2 (11.1%)
Other	1 (5.6%)
Not disclosed	2 (11.1%)
Length of work experience	
Less than 1 year	1 (5.6%)
1–2 years	0 (0.0%)
3–4 years	2 (11.1%)
5–6 years	3 (16.7%)
7–8 years	1 (5.6%)
9–10 years	0 (0.0%)
Over 10 years	11 (61.1%)
Education in perinatal bereavement support	
Yes	11 (61%)
No	7 (39%)

### 3.2. Participants’ Workplace Context and Current Practice

The survey asked some clarifying questions around how pregnancy loss care was organised in the participants’ clinical setting. A majority (94%) of participants said their place of work had a care pathway or guidelines for pregnancy loss care. Half of the participants (*n* = 9) reported they dealt with pregnancy loss on a daily basis in their work. In terms of the type of pregnancy loss for which participants had experience of providing care, all (*n* = 18) had experience of miscarriage (medical management), and *n* = 17 had experience of miscarriage (surgical management, expectant management and ectopic). Termination of pregnancy represented the area with the least staff experience, with *n* = 12 participants reporting experiences of medical management and *n* = 11 reporting experience of surgical management.

In terms of the clinical ward environment in which care was delivered, most participants reported that women who experience pregnancy loss are usually cared for in a private room with ensuite (*n* = 11), with six identifying women were cared for in a private room (no ensuite) and one in a Bay in an open ward. Sixteen (89%) confirmed that women experiencing pregnancy loss are able to have a partner or support person with them at all times, when an inpatient in the ward. The remaining two (11%) said a partner or a support person was only allowed to be in the clinical ward during the day, but not overnight.

Responses varied regarding how staff are allocated to care for someone going through pregnancy loss. Allocation to the most senior person on shift was identified by five participants. Allocation to someone who had interest or experience in bereavement care was identified by six participants, with a further six identifying random allocation. Participants were asked who they thought was the best placed staff member to provide care for families in certain related situations. The responses are presented in Table 3.

**TABLE 3:** Perceptions of most appropriate staff to provide care.

	Nurse	Midwife	Healthcare assistant	Doctor
Diagnosis and care planning/options	14 (77.8%)	2 (11.1%)	0 (0%)	2 (11.1%)
Physical care during hospital stay (excluding any procedure which needs to be carried out by specifically qualified staff)	11 (61.1%)	2 (11.1%)	2 (11.1%)	3 (16.7%)
Emotional support	15 (83.3%)	2 (11.1%)	1 (5.6%)	0 (0%)
Care of baby and memory making (if possible and wanted)	15 (83.3%)	2 (11.1%)	1 (5.6%)	0 (0%)
Discussion of options regarding baby’s remains	11 (61.1%)	2 (11.1%)	0 (0%)	5 (27.8%)
Aftercare	15 (83.3%)	2 (11.1%)	0 (0%)	1 (5.6%)

### 3.3. PBCCS

#### 3.3.1. Perinatal Bereavement Knowledge

Overall, participants recognised the significance of perinatal bereavement and the importance of professional support with 83.3% of respondents strongly agreeing that perinatal loss is a traumatic event for parents and 83.4% agreeing or strongly agreeing that bereaved parents require the support of HCPs. Similarly, 77.8% strongly agreed that grieving is a process, reflecting broad conceptual understanding of bereavement. However, respondents’ knowledge appeared less consistent in relation to specific care needs. While only one participant (5.6%) expressed not knowing how to provide bereavement support to the needs of grieving mothers, 27.8% (*n* = 5) did not feel confident providing support for the needs of grieving fathers. Reported understandings of cultural (66.7% agreed/strongly agreed), social (72.2% agreed/strongly agreed) and religious (55.6% agreed/strongly agreed) needs were also more variable.

In terms of practical knowledge, over a fifth of participants (22.2%) agreed that they lacked adequate practical bereavement knowledge. Furthermore, only a minority (11.1%) felt well prepared to provide perinatal bereavement support. Despite these gaps, there was near to universal recognition of the need for further training with 94.5% agreeing or strongly agreeing that ongoing perinatal bereavement education is necessary (See Table 4).

**TABLE 4:** Perinatal bereavement knowledge.

Statements	1	2	3	4	5
	Strongly disagree	Disagree	Neither agree/disagree	Agree	Strongly agree
Perinatal loss is a traumatic event for bereaved parents	1 (5.6%)	0 (0%)	0 (0%)	2 (11.1%)	15 (83.3%)
Bereaved parents require the support of HCPs to cope with their loss	1 (5.6%)	0 (0%)	2 (11.1%)	5 (27.8%)	10 (55.6%)
I understand that grieving is a process	1 (5.6%)	0 (0%)	1 (5.6%)	2 (11.1%)	14 (77.8%)
I know how to provide the specific bereavement support needs of grieving mothers	0 (0%)	1 (5.6%)	1 (5.6%)	12 (66.7%)	4 (22.2%)
I understand the cultural needs of bereaved parents	0 (0%)	4 (22.2%)	2 (11.1%)	7 (38.9%)	5 (27.8%)
I understand the social needs of bereaved parents	0 (0%)	2 (11.1%)	3 (16.7%)	8 (44.4%)	5 (27.8%)
I do not know the legal process associated with perinatal loss before 24 weeks gestation	3 (16.7%)	9 (50%)	2 (11.1%)	2 (11.1%)	2 (11.1%)
I do not know how to provide the specific bereavement support needs of grieving fathers	2 (11.1%)	8 (44.4%)	3 (16.7%)	3 (16.7%)	2 (11.1%)
I understand the religious needs of bereaved parents	0 (0%)	3 (16.7%)	5 (27.8%)	7 (38.9%)	3 (16.7%)
I know the referral system for additional bereavement support	0 (0%)	2 (11.1%)	0 (0%)	9 (50%)	7 (38.9%)
I do not have adequate practical knowledge for bereavement support	4 (22.2%)	7 (38.9%)	3 (16.7%)	4 (22.2%)	0 (0%)
I know the legal process associated with perinatal loss after 24 weeks gestation	1 (5.6%)	6 (33.3%)	1 (5.6%)	6 (33.3%)	4 (22.2%)
I have been well prepared to provide perinatal bereavement support	0 (0%)	6 (33.3%)	7 (38.9%)	3 (16.7%)	2 (11.1%)
There is a need for continuing perinatal bereavement education for HCPs.	0 (0%)	1 (5.6%)	0 (0%)	5 (27.8%)	12 (66.7%)
All HCPs at the hospital should receive perinatal bereavement education	0 (0%)	1 (5.6%)	3 (16.7%)	4 (22.2%)	10 (55.6%)

#### 3.3.2. Skills for Providing Perinatal Bereavement Support

Participants reported mixed levels of confidence in their skills for providing perinatal bereavement support. Just over 60% agreed or strongly agreed that they had the skills to provide practical support to recently bereaved parents, and 22.2% believed they lacked adequate bereavement support experience. Confidence in grief counselling skills was limited, with only 27.8% agreeing or strongly agreeing that they possessed these skills with the majority (72.3%) disagreeing or strongly disagreeing. In contrast, participants reported greater confidence in communication‐based support: 94.4% agreed or strongly agreed that they could listen comfortably to bereaved parents. 94.4% also felt able to provide emotional care. Less confidence was reported in having skills to provide spiritual support with 44.5% agreeing or strongly agreeing they could provide such care. Responses also highlighted lower confidence in addressing the needs of bereaved siblings (38.9% agreement) and parents expecting a subsequent baby (55.5% agreement) (See Table 5).

**TABLE 5:** Skills for providing perinatal bereavement support.

Statements	1	2	3	4	5
	Strongly disagree	Disagree	Neither agree/disagree	Agree	Strongly agree
I have the skills to provide practical support to recently bereaved parents	0 (0%)	2 (11.1%)	5 (27.8%)	7 (38.9%)	4 (22.2%)
I do not have adequate perinatal bereavement support experience	3 (16.7%)	8 (44.4%)	3 (16.7%)	3 (16.7%)	1 (5.6%)
I have grief counselling skills for providing psychological support to bereaved parents	3 (16.7%)	10 (55.6%)	0 (0%)	1 (5.6%)	4 (22.2%)
I can provide the relevant information required by bereaved parents	0 (0%)	2 (11.1%)	3 (16.7%)	8 (44.4%)	5 (27.8%)
I can comfortably listen to bereaved parents without trying to interrupt them.	0 (0%)	1 (5.6%)	0 (0%)	6 (33.3%)	11 (61.1%)
I can provide emotional care to bereaved parents	0 (0%)	1 (5.6%)	0 (0%)	8 (44.4%)	9 (50%)
I can provide spiritual care to bereaved parents	1 (5.6%)	5 (27.8%)	4 (22.2%)	5 (27.8%)	3 (16.7%)
I can easily respond to the needs of bereaved sibling when accompanying their parents	0 (0%)	5 (27.8%)	6 (33.3%)	4 (22.2%)	3 (16.7%)
I can easily respond to the needs of bereaved parents expecting their next baby	0 (0%)	4 (22.2%)	4 (22.2%)	6 (33.3%)	4 (22.2%)

#### 3.3.3. Self‐Awareness

Findings related to self‐awareness highlighted variable levels of confidence among participants. While 72.2% of respondents agreed or strongly agreed that they were aware of the needs of recently bereaved parents, fewer (55.6%) indicated confidence in recognising the particular needs of parents expecting a subsequent baby. Most participants reported the ability to empathise with grieving parents, with 83.3% agreeing or strongly agreeing with this statement. Awareness of personal limitations and learning needs was also common, with 83.3% of participants acknowledging these respective areas. However, engagement in reflective practice appeared less consistent, with only 50% reporting agreement or strong agreement. These results suggest that participants demonstrated strong empathic awareness and recognition of their learning needs, but fewer reported active engagement in reflective practice or awareness of the nuanced needs of parents in subsequent pregnancies (See Table 6).

**TABLE 6:** Self‐awareness.

Statements	1	2	3	4	5
	Strongly disagree	Disagree	Neither agree/disagree	Agree	Strongly agree
I am aware of the needs of recently bereaved parents	0 (0%)	3 (16.7%)	2 (11.1%)	9 (50%)	4 (22.2%)
I can easily empathise with grieving parents (empathy means that I can emotionally put myself in their place).	0 (0%)	2 (11.1%)	1 (5.6%)	7 (38.9%)	8 (44.4%)
I am conscious of the particular needs of bereaved parents expecting their next baby	0 (0%)	3 (16.7%)	5 (27.8%)	5 (27.8%)	5 (27.8%)
I am aware of my limitations in relation to the provision of perinatal bereavement support	0 (0%)	1 (5.6%)	2 (11.1%)	11 (61.1%)	4 (22.2%)
I am aware of my learning needs regarding bereavement support	0 (0%)	1 (5.6%)	2 (11.1%)	11 (61.1%)	4 (22.2%)
I am regularly engaged in reflective practice in relation to the provision of perinatal bereavement support	0 (0%)	3 (16.7%)	6 (33.3%)	7 (38.9%)	2 (11.1%)
I am aware of my personal resources for bereavement support	0 (0%)	2 (11.1%)	5 (27.8%)	7 (38.9%)	4 (22.2%)
Being aware of my need for support in relation to providing care for bereaved parents encourages me to seek help	0 (0%)	1 (5.6%)	6 (33.3%)	6 (33.3%)	5 (27.8%)

#### 3.3.4. Organisational Support

Responses relating to organisational support were varied. While two‐thirds of participants (66.7%) reported having support from workplace management in relation to bereavement care, less than half (44.4%) felt they received recognition for providing such support. Peer support was more consistently endorsed, with 83.3% agreeing or strongly agreeing that it was available in their workplace. Conversely, only 33.3% of respondents agreed or strongly agreed that their work environment allowed them to feel relaxed in carrying out daily work with fewer than one in five (16.7%) feeling that managers organised daily work placements to facilitate them to provide bereavement support. Workload was identified as a barrier to hindering the provision of effective bereavement (61.1% agreeing or strongly agreeing). Although 72.3% of respondents indicated that a clear policy for bereavement support was in place within their unit, only 22.2% felt there were sufficient staff to enable the delivery of such care. While 83.4% reported that their organisation provides bereavement support training, only 27.8% agreed or strongly agreed that debriefing opportunities were consistently available following traumatic incidents. Overall, the findings highlight the importance of peer networks but point to significant organisational constraints, particularly in relation to staffing, workload and structured support mechanisms (See Table 7).

**TABLE 7:** Organisational support.

Statements	1	2	3	4	5
	Strongly disagree	Disagree	Neither agree/disagree	Agree	Strongly agree
I have support from my workplace management in relation to providing bereavement support	1 (5.6%)	1 (5.6%)	4 (22.2%)	10 (55.6%)	2 (11.1%)
I have adequate peer support in my workplace in relation to providing bereavement support	0 (0%)	1 (5.6%)	2 (11.1%)	13 (72.2%)	2 (11.1%)
My work environment allows me to feel relaxed to carry out my daily work	1 (5.6%)	7 (38.9%)	4 (22.2%)	4 (22.2%)	2 (11.1%)
I get recognition for providing effective bereavement support	2 (11.1%)	4 (22.2%)	4 (22.2%)	7 (38.9%)	1 (5.6%)
The manager organises my daily work placements to facilitate me to provide bereavement support	5 (27.8%)	4 (22.2%)	6 (33.3%)	2 (11.1%)	1 (5.6%)
My workload hinders effective bereavement support.	1 (5.6%)	2 (11.1%)	4 (22.2%)	8 (44.4%)	3 (16.7%)
There is a clear policy in my ward/unit for the provision of bereavement support to parents	0 (0%)	3 (16.7%)	2 (11.1%)	10 (55.6%)	3 (16.7%)
There is adequate number of HCPs to cover the ward/unit to enable the provision of bereavement support.	5 (27.8%)	6 (33.3%)	3 (16.7%)	4 (22.2%)	0 (0%)
My organisation provides bereavement support training	0 (0%)	1 (5.6%)	2 (11.1%)	13 (72.2%)	2 (11.1%)
Debriefing opportunities are always provided for me when required following a traumatic incident.	4 (22.2%)	4 (22.2%)	5 (27.8%)	4 (22.2%)	1 (5.6%)
The workload of the ward/unit hinders effective bereavement support	0 (0%)	1 (5.6%)	6 (33.3%)	4 (22.2%)	7 (38.9%)

### 3.4. Qualitative Content Analysis

Analysis of the open‐text survey responses (*n* = 41) generated four overarching categories reflecting HCPs’ perspectives on pregnancy loss care within gynaecological settings: (1) creating safe and compassionate spaces, (2) systemic and structural pressures undermine care, (3) the need for skilled, confident and supported staff and (4) building organisational commitment to bereavement care. Cumulatively, the findings illustrate that HCPs aspire to deliver sensitive person‐centred bereavement care but are often hindered by structural and organisational constraints. Table 8 summarises the four overarching categories and corresponding subcategories identified through qualitative content analysis.

**TABLE 8:** Categories and subcategories derived from qualitative content analysis.

Category	Subcategories	Illustrative participant quotes
Creating safe and compassionate spaces	• Privacy and dignity • One‐to‐one, person‐centred care • Time with baby and family • Empathetic communication	‘All women and partners should have an allocated nurse and private room’. (HCP03) ‘A peaceful ward where there aren’t confused patients… time for the patient and their partner to spend with their baby after delivery’. (HCP07) ‘Listening, supporting, communicating well’. (HCP02)
Systemic and structural pressures undermine care	• Inappropriate mixed‐use environments • Staffing shortages and heavy workloads • Competing clinical priorities • Lack of managerial support	‘We are caring for pregnancy loss patients, elderly, immobile, confused and dying patients all in the same setting’. (HCP09) ‘Staffing is the main issue. Often the nurse looking after a woman experiencing a mid‐trimester loss is also responsible for six other patients’. (HCP06) ‘Nobody fights our corner in gynae’. (HCP10)
The need for skilled, confident and supported staff	• Knowledge and procedural confidence • Communication and counselling skills • Experiential learning (simulation, reflection) • Debriefing and emotional support	‘Regular training and updates with bereavement midwives… step‐by‐step guide of what to do with the baby once delivered’. (HCP05) ‘Debriefs following care of pregnancy loss patients would help us process what we’ve experienced’. (HCP12)
Building organisational commitment to bereavement care	• Investment in staffing and specialist roles • Clear policies and care pathways • Whole‐team bereavement awareness • Recognition of emotional impact	‘There aren’t enough bereavement midwives in the trust’. (HCP08) ‘All staff on the ward, domestics included, need appropriate training… it enables us to help and support parents’. (HCP04)

#### 3.4.1. Creating Safe and Compassionate Spaces

Participants described high‐quality pregnancy loss care as grounded in privacy, dignity and emotional presence. The availability of private rooms and one‐to‐one nursing was viewed as essential for enabling sensitive communication and uninterrupted time with the baby and family. Compassionate listening and continuity of care were seen as markers of good practice, allowing women and partners to feel acknowledged and supported during an intensely vulnerable period. As one participant explained, ‘*All women and partners should have an allocated nurse and private room’* (HCP03), reflecting a shared belief that the physical and emotional environment fundamentally shapes the experience of care.

#### 3.4.2. Systemic and Structural Pressures Undermine Care

Despite a personal commitment to compassionate practice, participants described significant systemic barriers that constrained their ability to deliver this consistently. Many worked on mixed‐use wards where women experiencing pregnancy loss shared space with elderly or medical patients. This was described as distressing for both families and staff, with one respondent noting, ‘*We are caring for pregnancy loss patients, elderly, immobile, confused and dying patients all in the same setting’* (HCP09). Chronic staffing shortages, high workloads and limited recognition or support from management were recurrent concerns, contributing to staff morale, distress and fatigue. These accounts highlight how environmental and organisational factors can compromise the quality of care and the commitment, safety and wellbeing of staff.

#### 3.4.3. The Need for Skilled, Confident and Supported Staff

All participants expressed a strong desire for enhanced training and professional support. Many felt that existing educational opportunities were too limited, inconsistent or overly theoretical. Respondents identified a number of training and support needs including regular multidisciplinary sessions covering communication, counselling and procedural aspects such as care of the baby’s remains. Simulation and reflective learning were suggested as practical approaches to improve confidence. The need for postevent debriefing was also emphasised as a means of promoting both professional growth and emotional wellbeing: ‘*Debriefs following care of pregnancy loss patients would help us process what we’ve experienced’* (HCP12).

#### 3.4.4. Building Organisational Commitment to Bereavement Care

The final category reflected participants’ recognition that sustainable improvement depends on organisational culture and leadership. Respondents called for greater investment in staffing levels, specialist bereavement roles and clear care pathways to ensure consistency and accountability. Extending bereavement awareness to all ward staff, including healthcare assistants and domestic workers, was viewed as crucial to delivering cohesive and compassionate care. As one participant stated, ‘*All staff on the ward, domestics included, need appropriate training… it enables us to help and support parents’* (HCP04). These insights suggest that while individual empathy is strong, systemic commitment remains inconsistent across services.

## 4. Discussion

This mixed‐methods study provides a novel insight into HCPs’ views about their own confidence and skills and their respective organisations’ support for pregnancy loss care in gynaecological settings in NI. Quantitative findings revealed that while participants have awareness of and empathy for bereaved women’s needs, many feel less confident of their skills and knowledge in providing bereavement support to fathers, in situations of cultural and religious diversity or legalities involved in perinatal loss after 24 weeks. Qualitative data reinforced these results, highlighting structural and systemic barriers such as staffing shortages, lack of dedicated bereavement spaces, lack of management support and inconsistent training.

The high proportion of respondents acknowledging pregnancy loss as a traumatic event reflects an empathetic workforce [27], yet gaps in procedural and psychosocial confidence mirror international evidence [18, 19]. Limited confidence in supporting fathers and understanding legal frameworks suggests ambiguity for staff in navigating nonclinical aspects of loss. This aligns with other studies [28–30], including Kalu et al. [25], who reported similar patterns among Irish midwives using the PBCCS. Although fathers have historically been marginalised within maternity discourse and service configuration [31, 32], both parents have consistently described inclusion of both partners as an essential component of pregnancy loss care [33–35]. These findings reinforce the need for bereavement care to adopt a family systems approach, rather than concentrating solely on maternal clinical needs [36, 37].

Qualitative findings further illustrate that environmental and organisational factors actively limit the capacity of even highly skilled and compassionate staff to provide effective and dignified bereavement care. The current working environment within the NHS presents multiple challenges, particularly mixed‐use wards accommodating elderly or medically unwell patients. This was repeatedly described and distressing for both staff and the families they care for, corroborating the findings of Heaney [16] and Galeotti [7], who highlight that the physical environment is not peripheral, but a primary determinant of the quality and meaning of care, profoundly shaping patient experiences. Participants consistently emphasised that one‐to‐one nursing, privacy and protected relational time are not enhancements but foundational environmental prerequisites for bereavement care that maintains dignity, agency and emotional safety. Furthermore, chronic workforce shortages place considerable pressure on HCPs, reducing the time they can spend with families and further narrowing the conditions within which compassionate practice can occur. In combination, environment, workload and organisational arrangements exert systemic pressure that structurally constrain good care, findings replicated across different healthcare contexts and settings [38–40].

Consistent with Yang et al. [41], organisational support emerged as a critical determinant of staff wellbeing and care quality. Although peer support was widely reported, formal mechanisms, such as debriefing, reflective supervision or support from designated bereavement leads, were limited. This mirrors Freeman et al. [21], who found that a culture of emotional containment rather than open reflection, persists in acute gynaecological services. McCreight [17] observed that gynaecology nurses in NI providing pregnancy loss care appeared to benefit from the opportunity to share their experiences and reflect on their professional skills and coping mechanisms in a meaningful way. McCreight further suggested there was a strong risk of nurses’ needs being marginalised by the hidden nature of the emotional labour [17]. The emotional demands of repeated exposure to loss highlight the need for trauma‐informed workplace cultures that validate and support staff as both caregivers and grievers [22]. Consequently, such constraints may also impact the ability of staff to prioritise their own wellbeing, leading to increased stress and potential reductions in the overall quality of care [42].

Participants’ near‐universal call for further education underscores the important role ongoing training has on staff bereavement care competence and confidence and is in line with previous studies [43–46]. Evidence suggests that structured, longitudinal training combining simulation, reflective practice and multidisciplinary discussion enhances both confidence and compassion fatigue resilience [24]. However, specialist training on bereavement care for healthcare staff is limited [29, 30]. A recently developed training programme for perinatal bereavement care [*The TEARDROP Workshop*] in Ireland including all types of pregnancy loss warrants further investigation as an intervention that could be applied in other contexts [47].

### 4.1. Practice and Policy Implications

This study provides contemporary evidence on the confidence, skills and support needs of HCPs delivering pregnancy loss care within gynaecology settings, a largely underexplored research area. While the findings are situated within the NI context, they echo growing calls across the UK and internationally for improved training, protected care environments and stronger organisational structures to support staff to deliver effective bereavement care for families.

These study findings reinforce that while HCPs demonstrate a clear awareness of the emotional impact of pregnancy loss, systemic and educational barriers continue to limit their ability to deliver the level of care to which they aspire. The results highlight the need for protected bereavement environments, consistent multidisciplinary training and structured emotional support for staff who are routinely exposed to pregnancy loss. Integrating these elements into clinical governance frameworks could enhance both the quality of care and staff wellbeing. At a system level, the study points to the importance of organisational investment in bereavement leadership roles, standardised training pathways and policy frameworks that recognise pregnancy loss care as a core component and responsibility of gynaecological services.

Ensuring access to protected bereavement environments is critical to preserving privacy, dignity and emotional safety for families experiencing loss. The provision of dedicated or side rooms, separated from general or medical wards, allows staff to deliver focussed compassionate, family‐centred care without the competing demands of other clinical priorities.

Education also emerged as a key enabler of high‐quality bereavement support. Regular, multidisciplinary training encompassing communication skills, cultural and spiritual competence and knowledge and understanding of legal and procedural requirements would help to build staff confidence and ensure consistency in practice. Extending bereavement awareness to all members of the ward team including healthcare assistants, doctors and clerical staff would further promote consistency and compassion across the care environment.

Equally important is the provision of structured emotional support for HCPs themselves. Opportunities for debriefing, reflective supervision and peer discussion can help mitigate the emotional toll or burnout of staff’s repeated exposure to pregnancy loss as well as help promote professional resilience.

### 4.2. Limitations

This study has several limitations, which are acknowledged as follows:

- Recruitment through professional and voluntary sector networks may have resulted in self‐selection bias. The small, self‐selected sample size (*n* = 18) limits the generalisability of the findings and may overrepresent HCPs with a particular interest or confidence in bereavement care.
- Due to the anonymous nature of the survey and the small sample size, information regarding individual clinical units was not collected or reported. Consequently, it was not possible to determine whether all gynaecology units across NI were represented within the sample.
- The study did not collect detailed information regarding participants’ previous clinical experience across different settings, nor where or when bereavement education had been obtained. Consequently, it was not possible to determine how prior professional experience may have influenced participants’ confidence, skills or perceived support needs.
- Staff experiencing the greatest workload pressure, who may also have distinct training or support needs, were less likely to participate, potentially limiting the breadth of viewpoints represented.
- The qualitative content analysis was based on written responses to open‐ended survey questions rather than in‐depth interviews. As such, opportunities for clarification or exploration of emerging issues were constrained and the richness of the data may be more limited than in interview‐based studies, which could have captured richer and more nuanced data.
- The cross‐sectional design captures participants’ confidence and perceptions at a single point in time, preventing assessment of how these may evolve following training or organisational change.

Despite these limitations, the use of a mixed‐methods approach provided valuable triangulation, enhancing the depth and credibility of the findings and offering a nuanced understanding of staff experiences and needs within gynaecological bereavement care.

### 4.3. Conclusions

This study offers an important contribution to understanding the needs and experiences of HCPs providing bereavement care to parents experiencing pregnancy loss within gynaecology settings. Participants demonstrated strong empathy and awareness of the emotional impact of pregnancy loss as well as identifying how their ability to deliver optimal, person‐centred care was often constrained by systemic pressures and limited educational preparation. Addressing these barriers requires leadership, organisational commitment to adequate staffing, dedicated bereavement spaces and access for staff to ongoing, multidisciplinary training and reflective support. Addressing these issues will not only enhance staff wellbeing and professional confidence but also improve the quality and consistency of bereavement care for families.

Future research should explore the impact of structured educational and environmental interventions across gynaecological and early pregnancy care pathways with the aim of identifying, developing and evaluating good practice that can be shared and implemented at regional and national levels.

## Author Contributions

Suzanne Heaney (SH): conceptualisation, methodology, formal analysis, investigation, data curation, writing–original draft, writing–review and editing, and project administration. Áine Aventin (ÁA): conceptualisation, methodology, formal analysis, writing–review and editing, and supervision. Martina Galeotti (MG): conceptualisation, methodology, investigation, formal analysis, data curation, writing–review and editing, project administration and funding acquisition.

## Funding

We gratefully acknowledge the Martha McMenamin Memorial Scholarships Trust, who awarded Dr Martina Galeotti a scholarship to carry out this work. The Martha McMenamin Memorial Scholarships Trust is in no way affiliated to this work or its findings.

## Conflicts of Interest

The authors declare no conflicts of interest.

## Data Availability

Data are not made publicly available as informed consent was not obtained for this at the time of data collection. Anonymised data relating to study analyses may be made available upon reasonable request to the corresponding author.
